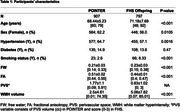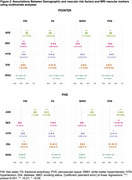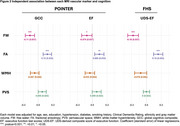# Risk factors and clinical relevance of MRI vascular markers in the U.S. POINTER study: a cross‐cohort comparison with the Framingham Heart Study

**DOI:** 10.1002/alz70856_100134

**Published:** 2025-12-25

**Authors:** Pauline Maillard, Prashanthi Vemuri, Heather M Snyder, Laura D Baker, Alexa S Beiser, Claudia L Satizabal, Jose Rafael Romero, Sudha Seshadri, Susan M. Landau, Charles Decarli, the US POINTER Study Group

**Affiliations:** ^1^ Department of Neurology and Center for Neuroscience, University of California, Davis, Davis, CA, USA; ^2^ Department of Radiology, Mayo Clinic, Rochester, MN, USA; ^3^ Alzheimer's Association, Chicago, IL, USA; ^4^ Wake Forest University School of Medicine, Winston‐Salem, NC, USA; ^5^ Boston University Chobanian & Avedisian School of Medicine, Boston, MA, USA; ^6^ Department of Biostatistics, Boston University School of Public Health, Boston, MA, USA; ^7^ Glenn Biggs Institute for Alzheimer's & Neurodegenerative Diseases, University of Texas Health Science Center, San Antonio, TX, USA; ^8^ The Framingham Heart Study, Framingham, MA, USA; ^9^ University of Texas Health San Antonio, San Antonio, TX, USA; ^10^ Neuroscience Department, University of California, Berkeley, Berkeley, CA, USA; ^11^ Department of Neurology & Imaging of Dementia and Aging Laboratory, University of California Davis, Sacramento, CA, USA

## Abstract

**Background:**

Our objective is to examine the generalizability of the relationships between four MRI markers of CVD: DTI‐derived free water (FW), fractional anisotropy (FA), perivascular space (PVS) and white matter hyperintensities (WMH) volume, with vascular risk factors (VRF) and cognition in the baseline POINTER Imaging and Framingham Heart Study (FHS) cohorts.

**Method:**

We studied 907 U.S. POINTER and 797 FHS Offspring participants with available baseline demographic, clinical and imaging data (Table 1). Continuous FW, FA, WMH (log‐transformed) and PVS measurements were regressed against total cranial volume (TCV). Cognitive outcomes included a Global Cognitive Composite and executive function composite score for POINTER and FHS. For each MRI marker, we used linear and logistic regressions to assess the effect of age, sex, hypertension, diabetes and smoking status, adjusting for ethnicity. We then assessed, for each cognitive outcome, the individual effect of MRI markers using separate linear regressions adjusted for demographic and clinical factors, as well as TCV‐adjusted gray matter volume to control for atrophy.

**Result:**

POINTER and FHS differed across most demographic and biomarker characteristics (Table 1). Despite these differences, there were similar associations between demographic and VRF and the 4 MRI vascular markers in POINTER and FHS (Figure 1): increased age was associated with worse outcomes for the 4 markers, hypertension was associated with increased FW, PVS and lower FA, diabetes was associated with increased FW only, smoking status did not impact any of the four markers. Only exception was that female had lower FW and increased WMH in POINTER and increased PVS in FHS. Associations between CVD measures and cognition were also similar across cohorts: reduced cognitive performance was significantly associated with both decreased FA and increased FW (Figure 2), but not with WMH nor PVS.

**Conclusion:**

Our study explores four imaging markers representing diverse mechanisms by which CVD could impact brain structure and function in the POINTER and FHS studies. Despite differences in cohort characteristics, we found mostly consistent CVD relationships with demographic, VRF, and cognitive measures between cohorts which supports the generalizability of the relation of these markers with VRF and cognition identified in the POINTER cohort.